# Ambra1 modulates the sensitivity of mantle cell lymphoma to palbociclib by regulating cyclin D1

**DOI:** 10.1038/s41598-023-35096-6

**Published:** 2023-05-24

**Authors:** Zhiping Jiang, Ao Zhang, Wenjia Wei, Shujun Li

**Affiliations:** 1 Department of Hematology, Xiangya Hospital, Central South University, Changsha, China; 2 National Clinical Research Center for Geriatric Diseases (Xiangya Hospital), Changsha, China; 3 Hunan Hematology Oncology Clinical Medical Research Center, Changsha, China

**Keywords:** Cancer, Cell biology

## Abstract

Mantle cell lymphoma (MCL) is a rare B-cell malignancy with a predominantly aggressive clinical course and poor prognosis. Abnormal expression of Ambra1 is closely related to the occurrence and development of various tumors. However, the role of Ambra1 in MCL remains unknown. Here, we performed both in vitro and in vivo experiments to investigate how Ambra1 regulates MCL progression and whether Ambra1 modulates the sensitivity of MCL cells to the CDK4/6 inhibitor palbociclib. We discovered that MCL cells had decreased levels of Ambra1 expression relative to normal B cells. Overexpression of Ambra1 in MCL cells inhibited autophagy, reduced cell proliferation, migration, and invasion, and decreased cyclin D1 level. While knockdown of Ambra1 reduced MCL cell sensitivity to CDK4/6 inhibitor palbociclib. Furthermore, overexpression of cyclin D1 lowered the sensitivity of MCL cells to palbociclib, enhanced cell proliferation, migration, invasion, and autophagy, and inhibited cell apoptosis. When Ambra1 expression was inhibited, the in vivo antitumor effects of palbociclib on MCL were reversed. Ambra1 expression was downregulated but cyclin D1 expression was upregulated in MCL samples, demonstrating a negative correlation between Ambra1 and cyclin D1. Our findings suggest a unique tumor suppressor function for Ambra1 in the development of MCL.

## Introduction

Mantle cell lymphoma (MCL) is a subtype of B-cell non-Hodgkin lymphoma that is characterized by a heterogeneous clinical presentation^1^. MCL accounts for approximately 3% to 10% of cases of non-Hodgkin lymphoma, and it is more common in male patients^2^. MCL is characterized by a characteristic chromosomal translocation, t(11;14)(q13;q32), which leads to overexpression of cyclin D1. Lymph node disease is common, especially in the gastrointestinal tract, spleen, and bone marrow^3^. The clinical course is largely aggressive, and the prognosis is poor, with high recurrence and mortality rates^3,4^. Nearly all patients eventually experience refractory disease or relapse, with a lethal sequence of drug resistance and continuous relapse, making treatment challenging^5^. Therefore, deepening the research and understanding of MCL pathogenesis can bring hope for the development of new targeted drugs.

Cyclin-dependent kinases (CDKs) are key molecules that regulate the cell cycle in mammals, and CDK4 and CDK6 are important members of the family^6^. After stimulation, CDK4/6 can bind to cyclin D to form a complex, and this cyclin D-CDK4/6 complex further phosphorylates the retinoblastoma (Rb) protein, making it unable to bind to the E2 transcription factor (E2F). As a result, E2F is activated and regulates cell cycle progression from G1 phase to S phase^7,8^. CDK4/6 is a key molecule involved in the development of various malignant tumors, and compared with pancycle inhibitor drugs, CDK4/6 inhibitors have stronger anticancer effects and less toxicity^9^. In addition, studies have shown that CDK4/6 is a very promising therapeutic target in treating MCL^10^.

Autophagy is the process by which damaged organelles or unnecessary proteins are encapsulated in a double-membrane structure and directed to lysosomes for degradation, a process that is particularly important for maintaining neuronal activity and normal cell function^11^. The autophagy function of normal cells is essential for disease resistance, but impaired autophagy leads to the occurrence of various diseases, such as neurodegenerative diseases, inflammatory diseases and cancers^12,13^. Autophagy is regulated by autophagy-related genes. Activating molecule in Beclin-1-regulated autophagy (Ambra1) is an activator of the autophagy-related gene Beclin-1. It binds to Beclin-1 and positively regulates the lipid kinase activity of the Beclin-1/VPS34 complex, thereby promoting autophagosome formation^14^. Studies have shown that Ambra1 deletion leads to increased cyclin D levels in cells and mice, promotes cell proliferation and reduces sensitivity to CDK4/6 inhibitors. Ambra1 deletion not only promotes lung adenocarcinoma growth, but a low level of Ambra1 is associated with poorer prognosis in lung adenocarcinoma patients^15^. In addition, abnormal Ambra1 expression is closely related to the occurrence and development of various tumors. However, the role of Ambra1 in MCL has not yet been reported.

Based on the above background, we explored the role of Ambra1 in the development of MCL and its internal regulatory mechanism through in vitro and in vivo experiments. Our research may provide new targets and ideas for the drug-resistant treatment of MCL.

## Materials and methods

### Collection of clinical samples

This research was carried out after all subjects provided informed consent and was approved by the Clinical Research Ethics Committee of Xiangya Hospital of Central South University (K202203149). Fifteen MCL patients and 15 healthy participants were included in our study. All MCL patients were confirmed by examination in the Department of Hematology, Xiangya Hospital of Central South University. Healthy control subjects were free of MCL at the time of physical examination. Blood samples were centrifuged at 3000 rpm for 10 min and stored at -80 °C until further processing.

### Cell treatment

IM-9 human peripheral blood B lymphocytes and MCL cell lines (Jeko-1, Z138, JVM2, and Rec-1) were purchased from Shanghai Zeye Biotechnology Co., Ltd. IM-9 cells were grown in RPMI-1640 medium supplemented with 10% FBS and 1% double antibiotic. Jeko-1, Z138, JVM2, and Rec-1 cells (all grown in suspension) were cultured in RPMI-1640 medium containing 10% FBS and 1% double antibiotic. The Ambra1 interference and overexpression vectors were constructed for transfection of JVM2 and Rec-1 cells or of Jeko-1 and Z138 cells, respectively. The groups were as follows: NC (oe-NC), ov-Ambra1; NC (si-NC), si-Ambra1#1, si-Ambra1#2, and si-Ambra1#3. A 2 μΜ concentration of the CDK4/6 inhibitor palbociclib^16^ or autophagy inhibitor chloroquine (CQ) was further added for 24 h. The cyclin D1 overexpression vector was constructed and transfected into Jeko-1 and Z138 cells. The ov-Ambra1, ov-cyclin D1, si-Ambra1 and corresponding negative control (NC) vectors were provided by Genepharma (Shanghai, China). The above sequences were transfected into cells using Lipofectamine 3000 (L3000150, Invitrogen) reagent.

### Quantitative real-time PCR (qRT‒PCR)

Total RNA was extracted by TRIzol (15596026, Thermo Fisher). cDNA was reverse transcribed using a cDNA reverse transcription kit (CW2569, CWBIO) with total RNA as a template. Ultra SYBR Mixture (CW2601, CWBIO) was used for qRT‒PCR experiments. GAPDH and the 2^-ΔΔct^ method were used to normalize gene expression. The primer sequences were as follows: Ambra1-F: GAGCAGGATCCAGAGAGCAC, Ambra1-R: CCTCTGGGCGTAGTATGCAG; cyclin D1-F: ACCTCTTCACCTTATTCATGGCT, cyclin D1-R: GCCTTTCCCGACCCTGCTAC; GAPDH-F: ACAGCCTCAAGATCATCAGC, GAPDH-R: GGTCATGAGTCCTTCCACGAT.

### Western blot

Total protein was extracted with RIPA lysis buffer (AWB0136, Abiowell), and the protein concentration was quantified by a BCA protein assay kit (BL521A, Biosharp) and mixed with SDS‒PAGE loading buffer. Proteins were transferred to a PVDF membrane after separation by gel electrophoresis, and the membrane was blocked with 5% skim milk solution and incubated with antibodies specific for LC3-II/I (14600-1-AP, 1: 2000, Proteintech), Beclin1 (11306-1-AP, 1: 1000, Proteintech), ATG5 (10181-2-AP, 1: 1000, Proteintech), p62 (18420-1-AP, 1: 1000, Proteintech), cyclin D1 (26,939–1-AP, 1: 1000, Proteintech), and β-actin (66009-1-Ig, 1: 1000, Proteintech) at 4 ℃ overnight. Then, the membrane was incubated with secondary antibodies. The membrane was incubated with ECL solution (AWB0005, Abiowell)for 1 min, and images were acquired in the imaging system (Supplementary material).

### Immunofluorescence (IF)

Suspended cells were evenly distributed by pipetting, and a small amount of the suspension was then smeared on the slide and left to dry. Triton X-100 (0.3%) was used for 30 min at 37 °C for permeabilization. Then, 5% BSA was used for blocking at 37 °C for 60 min. A suitable dilution of the anti-LC3 primary antibody (14600-1-AP, 1:100, Proteintech) was added dropwise onto the slide for overnight incubation at 4℃. Then, the slide was incubated with 50–100 μL goat anti-rabbit IgG (H + L) Cross-Adsorbed Secondary Antibody Alexa Fluor 488 (A-11008, 1:200, Thermo Fisher) at 37 °C for 90 min. DAPI working solution was added for nuclear staining at 37 °C for 10 min. The slides were sealed with buffered glycerin and observed under a fluorescence microscope (BA410T, MOTIC).

### Cell counting Kit-8 (CCK-8) assay

A total of 2 × 10^4^ cells were centrifuged in a 1.5 mL sterile centrifuge tube to obtain a cell pellet, and the cells were resuspended in the corresponding drug-containing medium according to the grouping configuration and then seeded in a 24-well plate. After 6, 12, and 24 h of treatment according to the group designations, a reaction solution containing 20% CCK-8 reagent was prepared, and 300 μL of the CCK-8 reaction solution was added to each well. After incubation at 37 °C and 5% CO_2_ for 4 h, the reaction solution was transferred to a 96-well plate, and the absorbance at 450 nm was measured on a Bio-Tek microplate reader.

### Transwell assays

For migration experiments, the lower compartment of the Transwell chamber was filled with 500 μL of 10% FBS complete medium. The cells were digested with trypsin to form a single-cell suspension, which was then diluted to a density of 1 × 10^6^ cells/mL in serum-free medium; 100 μL of this suspension was added to each well. The plate was placed in a 37 °C incubator for 48 h. The upper insert was removed, and the cells in the lower compartment were aspirated and counted by flow cytometry. For invasion experiments, a sterile pipette tip, an EP tube, Matrigel and a Transwell chamber were incubated at 4 °C overnight for precooling one day before the experiment. Then, 100 μL of ice-cold, serum-free DMEM was added to each well to dilute the Matrigel to a final concentration of 200 μg Matrigel per well. Then, the cells were incubated at 37 °C for 30 min, and we aspirated the supernatant. The subsequent steps were the same as those used for the migration experiments.

### Apoptosis assay

Cells treated as described above were directly collected by centrifugation, washed twice with PBS, and centrifuged at 2000 rpm for 5 min each time to collect approximately 3.2 × 10^5^ cells. Five hundred microliters of binding buffer, 5 μL of Annexin V-APC, and 5 μL of propidium iodide were used. The reaction was performed for 10 min at room temperature in the dark. Within 1 h, flow cytometry (A00-1-1102, Beckman) was used for observation and detection.

### Construction of a mouse MCL xenograft model

All animal experiments were approved by the Ethics Committee for Animal Experimentation of Xiangya Hospital of Central South University (Prot. ID 202203003). All methods are reported in accordance with ARRIVE guidelines. Beginning on the day before transplantation, 40 male CB17 SCID mice were intraperitoneally injected weekly with 0.2 mg of an anti-mouse interleukin 2 receptor beta monoclonal antibody (TMβ1) to eliminate natural killer cells. Then, the mice were randomly divided into two groups, and 4 × 10^7^ treated Jeko-1 cells were injected through the tail vein in a volume of 50 μL. The corresponding cell lines were inoculated in different groups: si-control group and si-Ambra1 group, 20 mice in each group. After injection, detailed observations and recordings were performed twice a week^17,18^. Then, each large group was randomly divided into two groups: si-control group and si-control + palbociclib group; si-Ambra1 group and si-Ambra1 + palbociclib group. Each group contained 10 mice. Fifteen days after cell injection, the mice were treated with or without palbociclib (3 mg/100 g body weight in a volume of 1 mL) by daily gavage^19^. Four weeks’ post-injection, the experiment was terminated. Mice were sacrificed by intraperitoneal injection of 150 mg/kg sodium pentobarbital.

### Statistical analysis

GraphPad Prism 8.0 software was used for statistical analysis. Student's t test or one-way ANOVA was used for comparisons between two or among multiple groups, respectively. Pearson correlation analysis was performed to assess the correlation between Ambra1 and cyclin D1 expression in MCL patients. *P* < 0.05 indicated that the difference was statistically significant.

### Ethics approval statement

The study followed the principles set forth in the World Medical Association Declaration of Helsinki and was approved by the Institutional Ethics Committee of Xiangya Hospital of Central South University (K202203149). All animal experiments have been approved by the Ethics Committee for Animal Experimentation of Xiangya Hospital of Central South University (Prot. ID 202203003). We confirm that informed consent was obtained from all from all subjects (and/or their legal guardian(s) included in the study.

## Results

### Overexpression of Ambra1 inhibited autophagy in MCL cells

**Figure 1:**
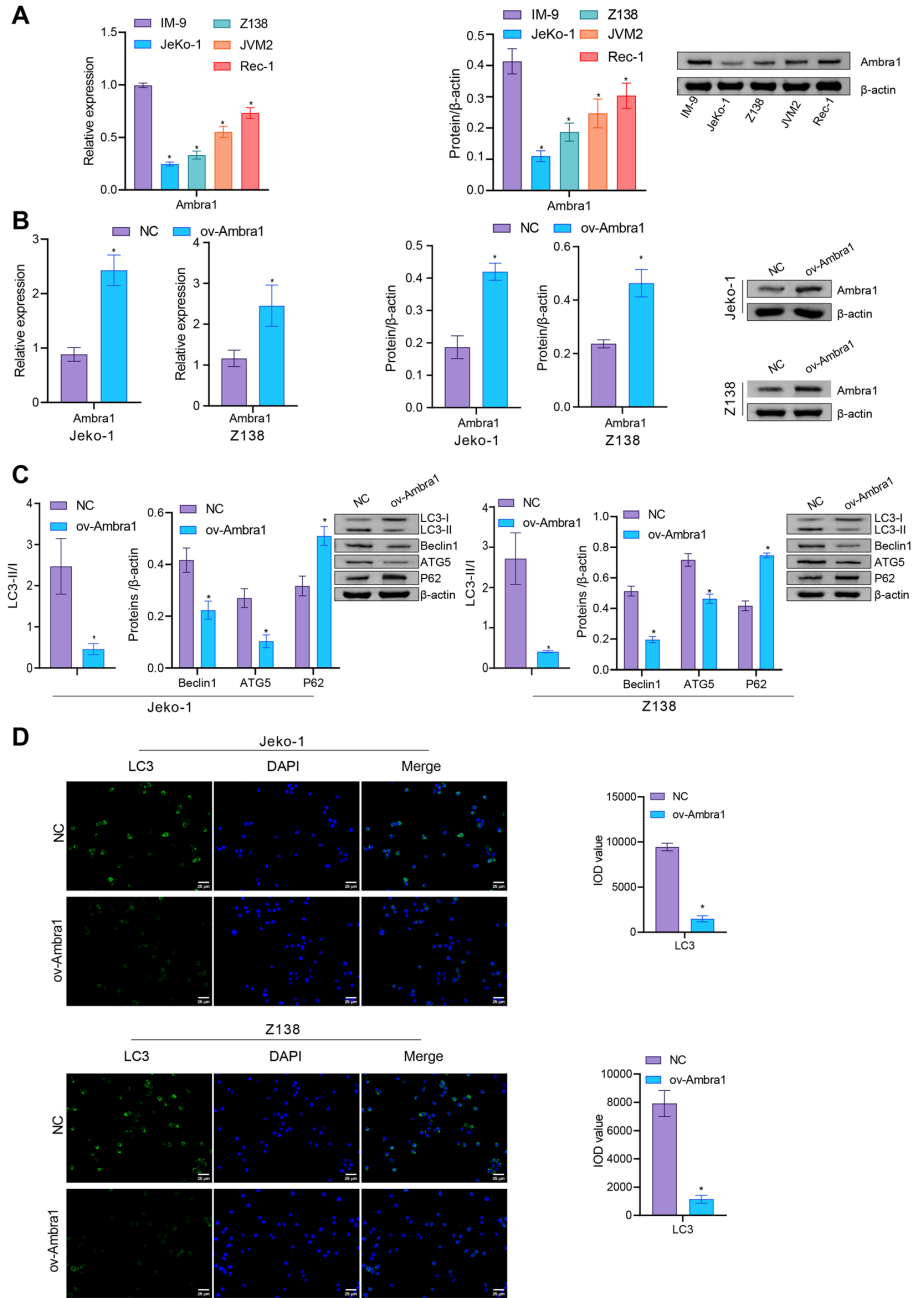
Overexpression of Ambra1 inhibited autophagy in MCL cells. (**A**) Ambra1 mRNA expression in MCL cell lines. *P < 0.05 versus IM-9 cells. After transfection of MCL cells with ov-Ambra1, (**B**) qRT‒PCR and western blot analyses of Ambra1 expression were performed. (**C**) Western blot analysis of LC3-II/I, Beclin 1, ATG5 and p62. (**D**). IF staining of LC3. Scale bar: 25 μm. n = 3. * *P* < 0.05 versus NC.

First, we measured Ambra1 expression in MCL cells. Compared with that in IM-9 human peripheral blood B lymphocytes, Ambra1 expression in MCL cells (Jeko-1, Z138, JVM2, and Rec-1) was significantly decreased. Among the MCL cell lines, Ambra1 expression was lower in Jeko-1 and Z138 cells (Fig. 1A). Next, we selected the two MCL cell lines with lower Ambra1 expression, Jeko-1 and Z138, for follow-up studies. We constructed an ov-Ambra1 plasmid to transfect Jeko-1 and Z138 cells and verified Ambra1 expression levels by qRT‒PCR and western blotting. We found that in Jeko-1 and Z138 cells, the Ambra1 levels were notably increased after overexpression of Ambra1 (Fig. 1B). Evaluation of autophagy-related factors showed that the LC3-II/I, Beclin-1 and ATG5 levels were significantly reduced after overexpression of Ambra1, while the p62 level was elevated (Fig. 1C and D). This suggested that overexpression of Ambra1 inhibited autophagy in MCL cells.

### Ambra1 overexpression affected the cyclin D1 level; inhibited proliferation, migration and invasion; and promoted apoptosis in MCL cells

**Figure 2:**
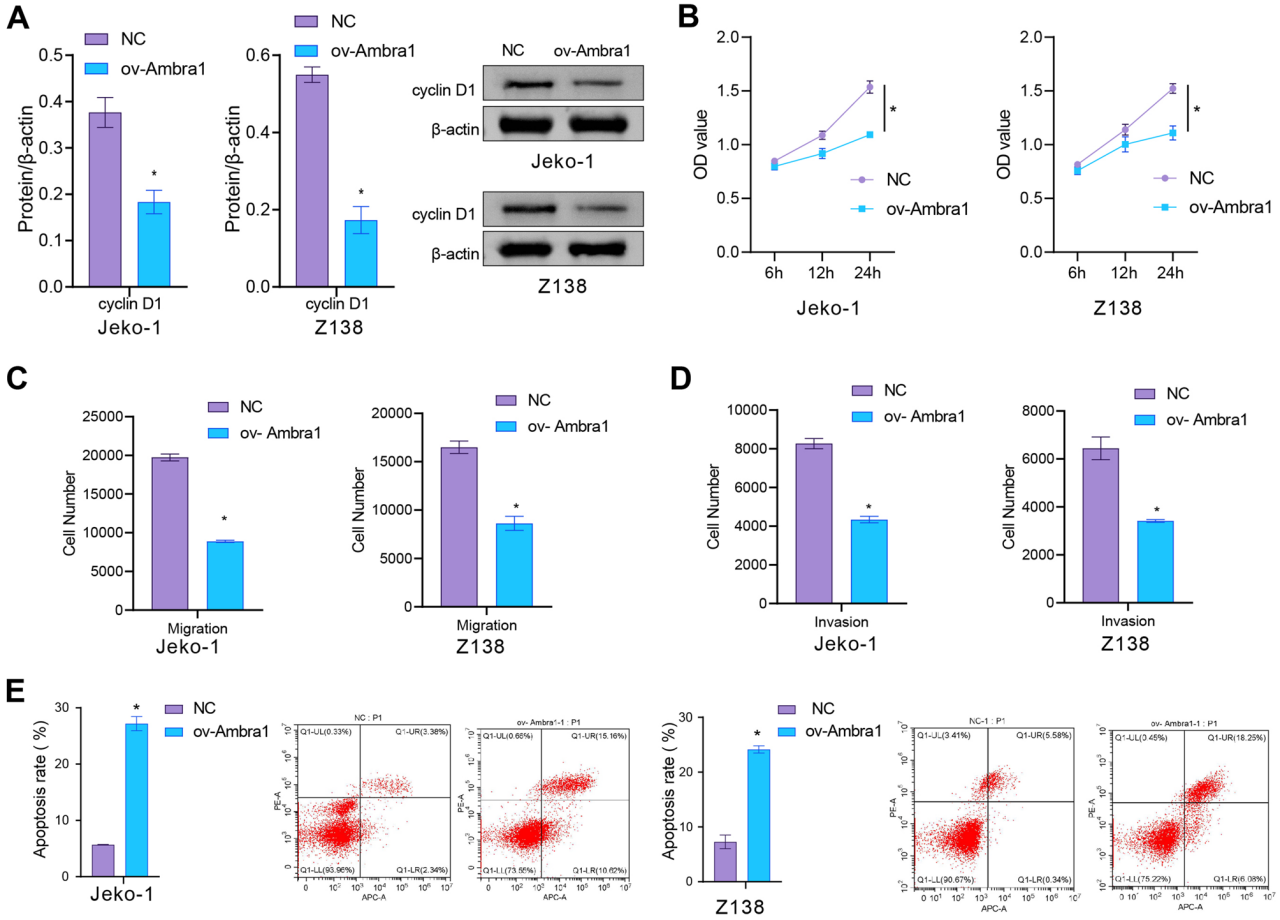
Ambra1 overexpression affected the cyclin D1 level; inhibited proliferation, migration and invasion; and promoted apoptosis in MCL cells. After transfection of MCL cells with ov-Ambra1, (**A**) cyclin D1 expression was measured. (**B**) Cell proliferation was assessed by a CCK-8 assay. (**C**) and (**D**) The migration and invasion of MCL cells. (**E**) Apoptosis in MCL cells. n = 3. * *P* < 0.05 versus NC.

Next, we examined the cyclin D1 level. We found that overexpression of Ambra1 decreased the cyclin D1 level in MCL cells (Fig. 2A). Cell functional experiments showed that compared with the NC group, MCL cells in the ov-Ambra1 group exhibited a decreased proliferation ability, decreased migration and invasion abilities, and increased apoptosis (Fig. 2B–E). These findings showed that Ambra1 could decrease the aggressive capacity of MCL cells and suppress the expression of cyclin D1.

### Ambra1 deletion reduced MCL cell sensitivity to the CDK4/6 inhibitor palbociclib

**Figure 3:**
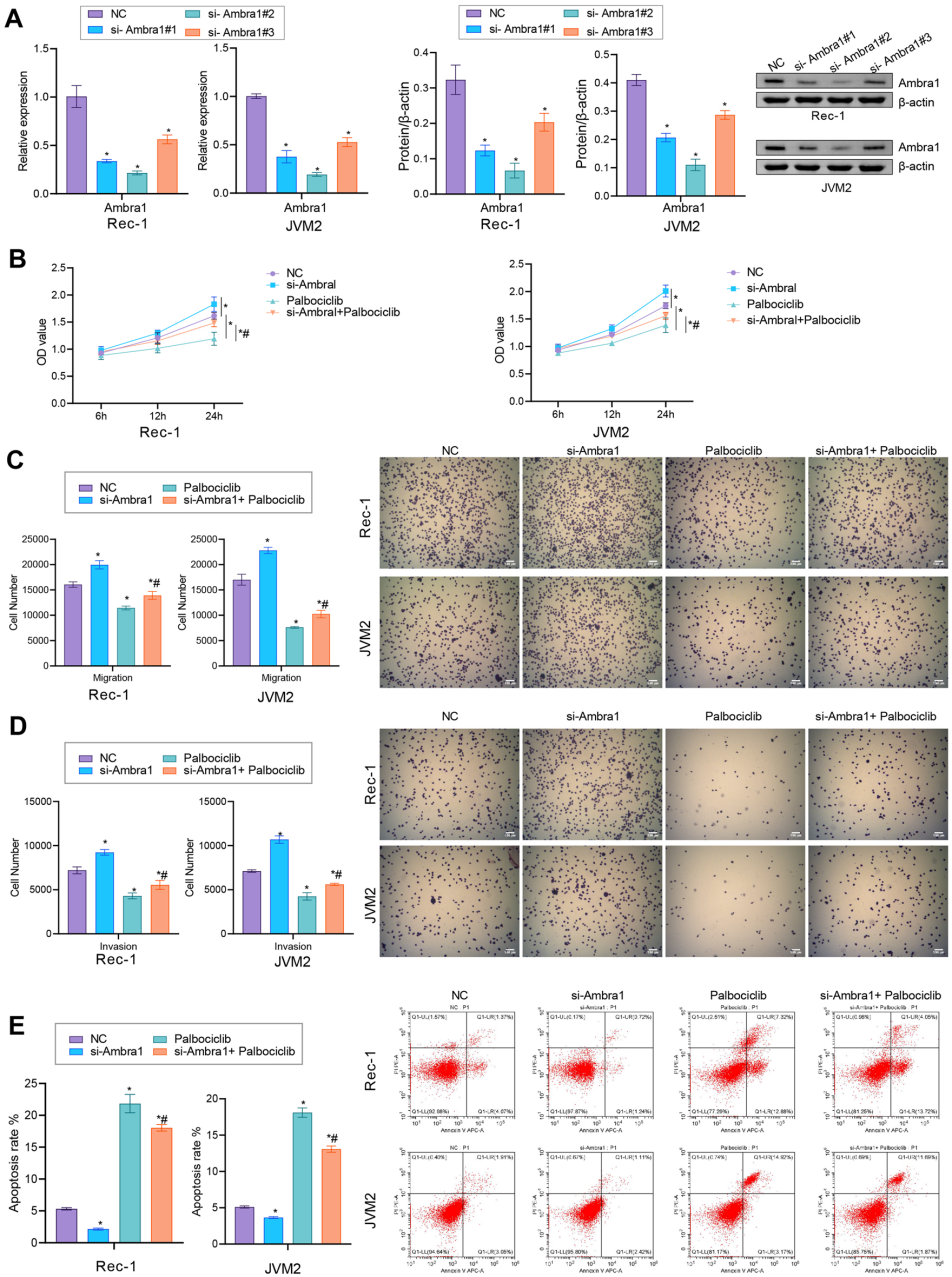
Ambra1 deletion reduced MCL cell sensitivity to the CDK4/6 inhibitor palbociclib. (**A**) After transfection of MCL cells with si-Ambra1, qRT‒PCR and western blot analyses of Ambra1 expression were performed. After treatment with or without si-Ambra1 or palbociclib, (**B**) cell proliferation was assessed by a CCK-8 assay. (**C**) and (**D**) The migration and invasion of MCL cells. (**E**) Apoptosis in MCL cells. n = 3. * *P* < 0.05 versus NC. # *P* < 0.05 versus palbociclib.

To further verify the biological impact of Ambra1 on the proliferation, migration, and invasion of MCL cells, we used shRNA to knock down Ambra1 expression in two MCL cell lines, Rec-1 and JVM2 (Fig. 3A). Compared with the NC group, Ambra1 expression in the si-Ambra1#2 group was significantly reduced. Additionally, we added the CDK4/6 inhibitor palbociclib. We found that in MCL cells, the proliferation ability was decreased, the migration and invasion abilities were decreased, and apoptosis was increased after adding palbociclib. However, these changes in cellular functions were reversed by deletion of the Ambra1 gene. That is, compared with the palbociclib group, the si-Ambra1 + palbociclib group had and increased proliferation ability, enhanced migration and invasion abilities, and decreased apoptosis (Fig. 3B–E). These findings suggested that Ambra1 affected MCL cell development and sensitivity to palbociclib in vitro.

### Ambra1 deletion partially abrogated the inhibitory effect of palbociclib on autophagy and increased the intracellular cyclin D1 level

**Figure 4:**
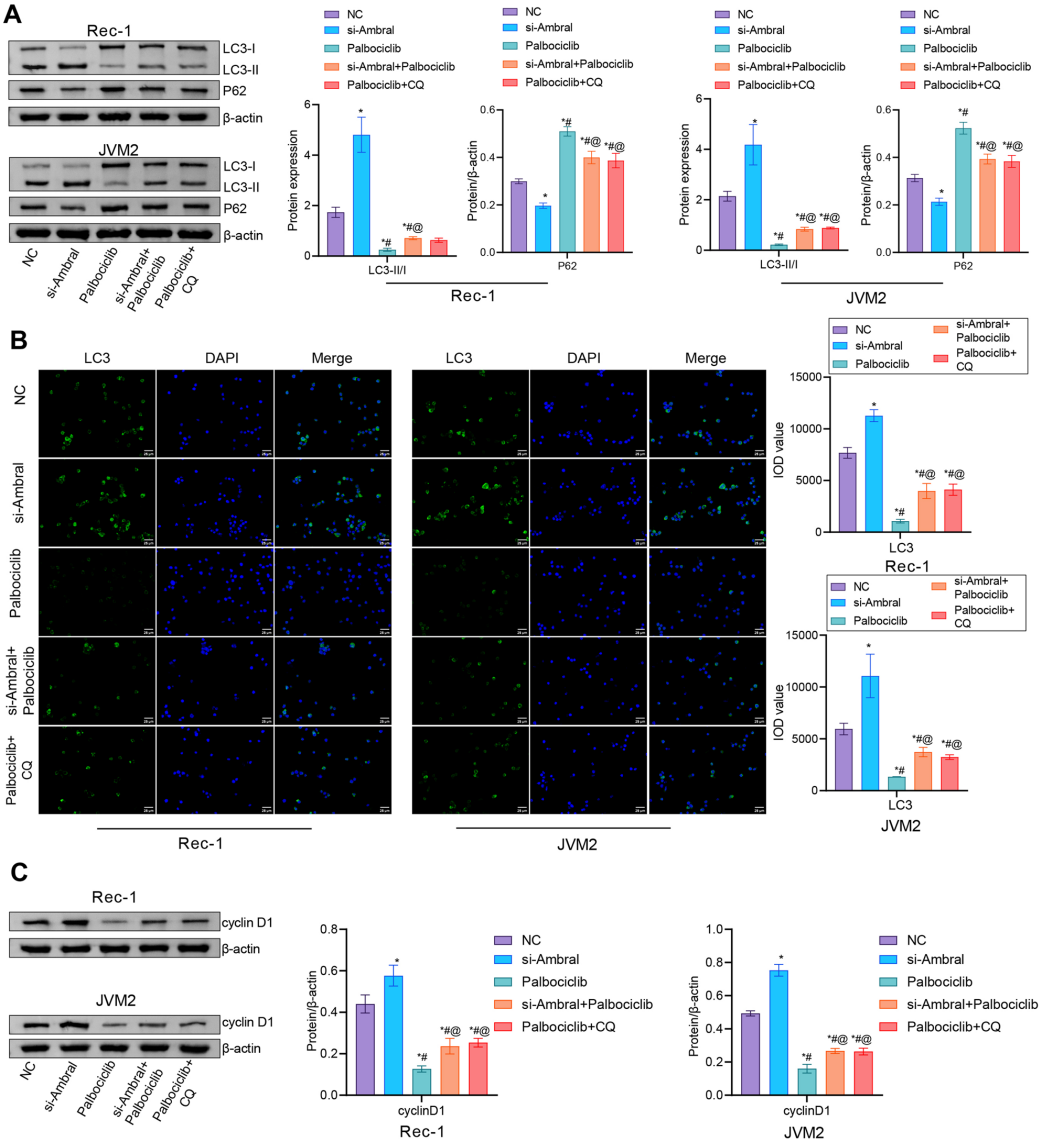
Ambra1 deletion partially abrogated the inhibitory effect of palbociclib on autophagy and increased the intracellular cyclin D1 level. (**A**) Western blot analysis of LC3-II/I and p62 in MCL cells exposed to 2 μM palbociclib, with or without simultaneous silencing of Ambra1 (si-Ambra1). (**B**) IF staining of LC3. Scale bar: 25 μm. (**C**) Cyclin D1 level in MCL cells. n = 3. * *P* < 0.05 versus NC. # *P* < 0.05 versus palbociclib.

Subsequently, we added the autophagy inhibitor CQ as a positive control group for research. After interference with Ambra1 expression, the LC3-II/I levels were significantly increased, while the p62 level was decreased. The levels of LC3-II/I was decreased but the p62 level was increased after adding palbociclib. However, these expression changes were reversed by deletion of the Ambra1 gene (Fig. 4A and B). In addition, the cyclin D1 level was increased after interference with Ambra1 expression. The cyclin D1 level was decreased after palbociclib treatment. Deletion of Ambra1 reversed the inhibition of intracellular cyclin D1 expression mediated by palbociclib (Fig. 4C). These results indicated that Ambra1 inhibition partially abrogated the inhibitory effect of palbociclib on autophagy and increased the intracellular cyclin D1 level.

### Overexpression of cyclin D1 reduced MCL cell sensitivity to palbociclib, facilitated MCL cell proliferation, migration, invasion and autophagy, and suppressed MCL cell apoptosis

**Figure 5:**
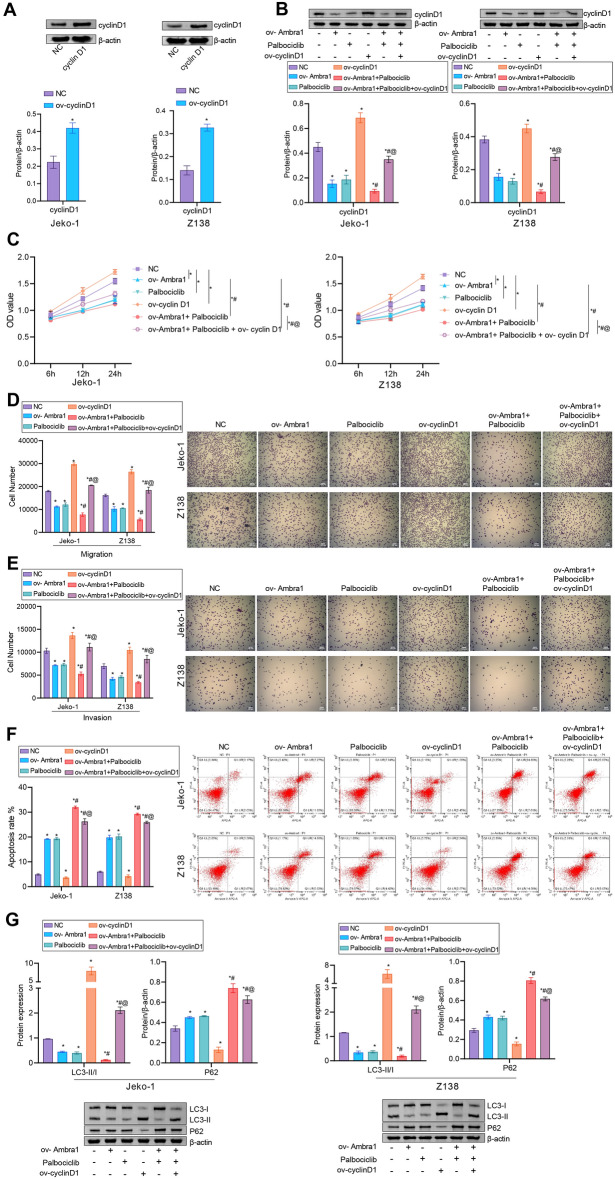
Overexpression of cyclin D1 reduced MCL cell sensitivity to palbociclib, facilitated cell proliferation, migration, invasion and autophagy, and suppressed apoptosis. (**A**) and (**B**) After transfection of MCL cells with ov-cyclin D1, western blot analysis of cyclin D1 expression was performed. After treatment with or without ov-cyclin D1 or palbociclib, (**C**) cell proliferation was assessed by a CCK-8 assay. (**D**) and (**E**) The migration and invasion of MCL cells. (**F**) Apoptosis in MCL cells. (**G**) Western blot analysis of LC3-II/I and p62 in MCL cells. n = 3. * *P* < 0.05 versus NC. # *P* < 0.05 versus palbociclib, @ *P* < 0.05 versus ov- Ambra1 + palbociclib.

To determine whether increased expression of cyclin D1 is responsible for MCL development and enhances resistance to the CDK4/6 inhibitor palbociclib, Jeko-1 and Z138 cells were transfected with the cyclin D1 overexpression vector or control vector. Overexpression of cyclin D1 significantly increased the cyclin D1 level (Fig. 5A). The cyclin D1 level was decreased after palbociclib treatment. Overexpression of cyclin D1 reversed the decrease in the cyclin D1 level in MCL cells mediated by palbociclib (Fig. 5B). Cell functional experiments showed that overexpression of cyclin D1 facilitated MCL cell proliferation, migration and invasion and suppressed apoptosis. After palbociclib treatment, the proliferation ability of MCL cells was decreased, the migration and invasion abilities were decreased, and apoptosis was increased. However, these cellular functions were reversed by overexpression of cyclin D1 (Fig. 5C–F). Figure 5G showed that overexpression of cyclin D1 increased the LC3-II/I levels and decreased the p62 level. After palbociclib treatment, the LC3-II/I levels were significantly decreased, while the p62 level was increased. However, these expression changes were reversed by overexpression of cyclin D1. In general, overexpression of cyclin D1 reduced MCL cell sensitivity to palbociclib; facilitated cell proliferation, migration, invasion and autophagy; and suppressed apoptosis.

### Ambra1 deficiency reversed the effect of palbociclib in vivo

**Figure 6:**
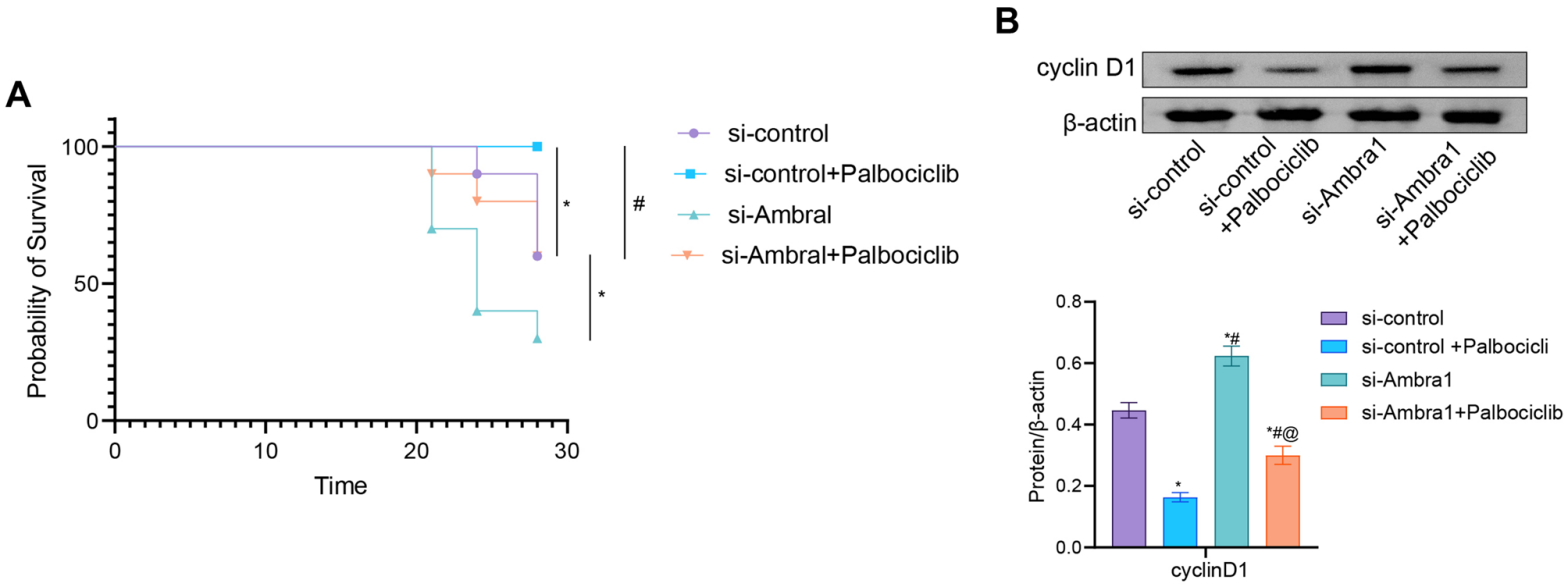
Ambra1 deficiency reversed the effect of palbociclib in vivo. (**A**) Survival probability of mice. (**B**) The cyclin D1 level was assessed by Western blotting. n = 10 mice/group. * *P* < 0.05 versus si-control. # *P* < 0.05 versus si-control + palbociclib.

Next, the impact of Ambra1 on sensitivity to palbociclib in vivo was investigated in a xenograft nude mouse model. Nude mice were injected with stable Jeko-1 cells expressing Ambra1 shRNA or control Jeko-1 cells transfected with the empty shRNA. Once tumors were visible, the mice were randomly separated into two groups and given either vehicle or palbociclib. As anticipated, Ambra1 silencing decreased the survival of mice relative to that of mice in the control group (Fig. 6A). We found that the survival rate of mice in the palbociclib group was significantly higher than that of mice in the vehicle group (Fig. 6A). When the expression of Ambra1 was downregulated, the effects of palbociclib on mouse survival were significantly attenuated (Fig. 6A). After animals were sacrificed, splenocytes were extracted, and lysates were immediately prepared to assess cyclin D1 levels using western blotting. The results demonstrated that treatment with palbociclib led to a significant decrease in cyclin D1 expression, whereas silencing Ambra1 induced cyclin D1 expression (Fig. 6B). These results suggested that the in vivo antitumor effects of palbociclib are reversed by Ambra1 deficiency.

### Reduced Ambra1 expression and elevated cyclin D1 expression in MCL patients

**Figure 7:**
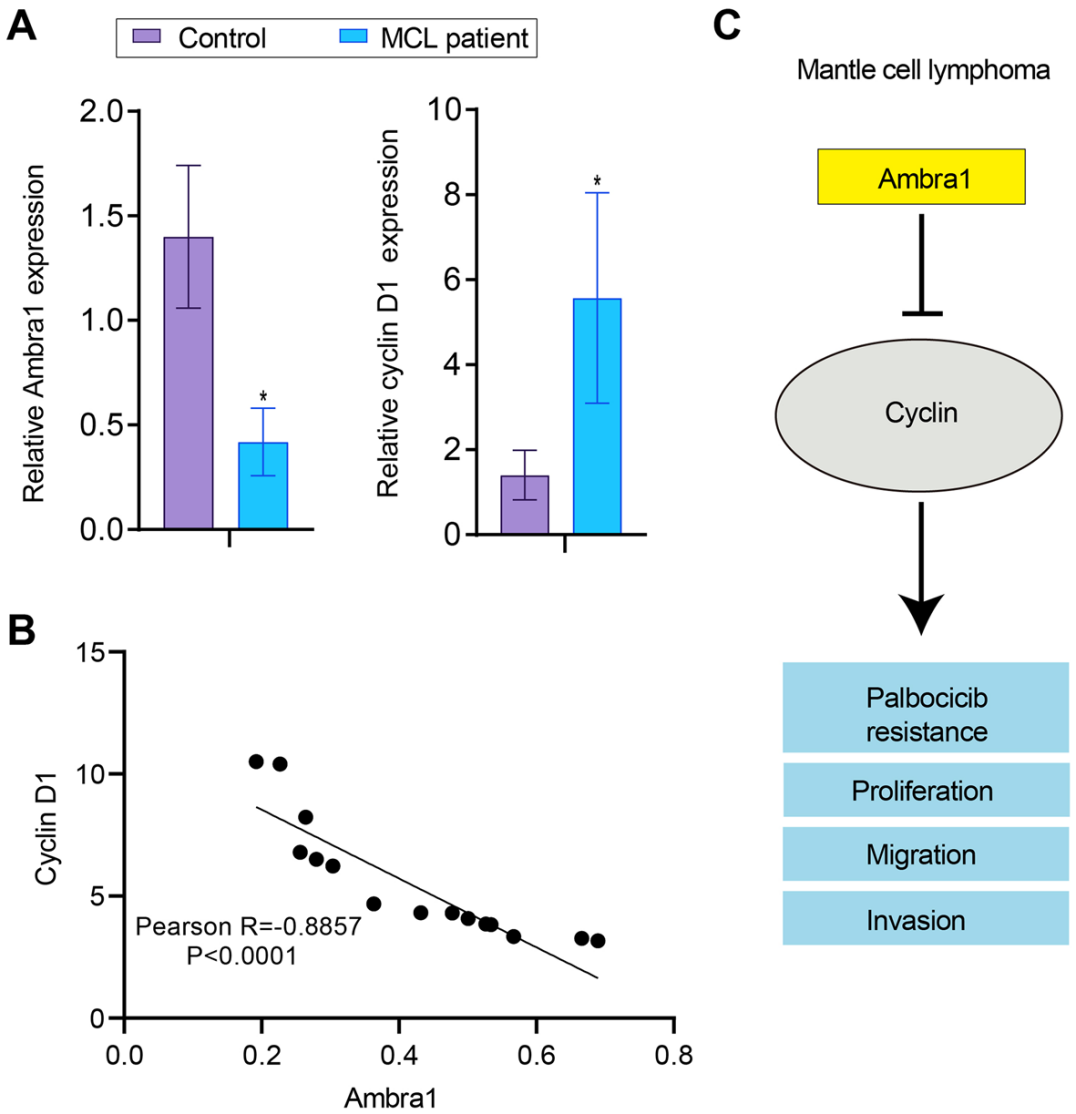
Reduced Ambra1 expression and elevated cyclin D1 expression in MCL patients. (**A**) The mRNA expression of *Ambra1* and *cyclin D1* in MCL samples and healthy participants (control) was measured using qRT‒PCR. (**B**) Pearson correlation analysis of the correlation between the *Ambra1* and *cyclin D1* mRNA levels measured using qRT‒PCR. n = 15. (**C**) The function and mechanisms of Ambra1 in MCL. Ambra1 deficiency increases the cyclin D1 level, which enhances MCL cell proliferation, migration, and invasion while decreasing sensitivity to the CDK4/6 inhibitor palbociclib. ***P < 0.001.

Using qRT‒PCR, we investigated the levels of *Ambra1* and *cyclin D1* in clinical samples. In MCL patients, *Ambra1* expression was significantly downregulated and cyclin D1 expression was significantly increased compared with the control group (Fig. 7A). Using Pearson correlation analysis, we discovered that *Ambra1* expression was negatively correlated with *cyclin D1* expression (Fig. 7B). Our findings support a model in which Ambra1 suppresses cyclin D1 expression and thereby overcomes palbociclib resistance in MCL (Fig. 7C).

## Discussion

Mantle cell lymphoma (MCL) is a rare but aggressive B-cell hematological malignancy characterized by the t(11;14)(q13;q32) translocation resulting in overexpression of cyclin D1^20^. Cell cycle dysregulation caused by aberrant cyclin D1 and CDK4 expression is a major determinant of cancer cell proliferation in MCL. Inhibition of CDK4/6 induces G1 arrest in patient MCL cells, which appears to enhance and prolong the clinical response to partner agents^21^. Ambra1 is an important regulator of embryonic development, and its mutation or inactivation has been shown to be associated with several pathologies of the nervous system and involved in carcinogenesis^22^. However, the role of Ambra1 in MCL has not yet been reported. In this study, we found through in vitro and in vivo studies that abnormal Ambra1 expression affected the cyclin D1 level and interfered with the sensitivity of MCL cells to the CDK4/6 inhibitor palbociclib. Reversing abnormal Ambra1 expression could inhibit the occurrence and development of MCL. Thus, evaluation of Ambra1 expression in MCL might be crucial to select MCL patients who may benefit from the administration of CDK4/6 inhibitors.

Autophagy is a recycling process highly conserved in eukaryotic cells, and dysfunction of this process leads to many disease pathologies^23^. Ambra1 is considered to be an autophagy and transport protein that plays a role in neurogenesis and cancer cell invasion^24^. Ambra1 can coordinate cellular responses to stress conditions such as starvation, and it is actively involved in cell proliferation, cytoskeleton modification, apoptosis, mitochondrial removal, and cell cycle downregulation^25^. In melanoma, Ambra1 deficiency accelerated tumor growth and reduced overall survival in a Braf/Pten mutant mouse model of melanoma. Furthermore, that study demonstrated that Ambra1 deletion promotes melanoma invasiveness and metastasis by increasing cell motility/invasion^26^. In addition, Ambra1 has been shown to modulate paclitaxel-induced apoptosis in breast cancer cells through the Bim/mitochondrial pathway, thereby altering the sensitivity of cells to paclitaxel^27^. Importantly, Zhao B et al. revealed that Ambra1 acts as a tumor suppressor, and its expression is reduced in uveal melanoma^28^. These studies suggest that Ambra1 plays a dual role in diseases. Consistent with the study of Zhao B et al., our results suggest that Ambra1 is a potential tumor suppressor in MCL. We found that the Ambra1 gene exhibited lower expression in MCL cells. Moreover, in MCL cells, overexpression of Ambra1 inhibited autophagy, affected the cyclin D1 level, inhibited proliferation, migration and invasion, and promoted apoptosis.

A study by Liu J et al. in prostate cancer showed that Ambra1 knockdown increased cisplatin-induced apoptosis. Furthermore, cisplatin-induced autophagy was upregulated by Ambra1 overexpression or downregulated by Ambra1 knockdown in prostate cancer cells^29^. Chaikovsky AC et al. reported that loss of AMBRA1 results in high levels of cyclin D in cells and in mice, which promotes proliferation and decreases sensitivity to CDK4/6 inhibition^15^. Consistent with the study of Chaikovsky AC et al., we also found that Ambra1 deletion reduced MCL cell sensitivity to the CDK4/6 inhibitor palbociclib. In vivo, Ambra1 deficiency reversed the effect of palbociclib. In MCL cells, cyclin D1 binds to CDK4/6 to phosphorylate Rb, releasing the block of G1 to S phase transition. Palbociclib is a specific and potent oral CDK4/6 inhibitor that induces complete, prolonged G1 arrest (pG1) in Rb + MCL cells^30^. In addition, inhibition of the master regulator of cellular metabolism, the protein kinase mTOR, induces autophagy and inhibits cell proliferation^31^. After further addition of the autophagy inhibitor CQ, we found that Ambra1 deletion partially abrogated the inhibitory effect of palbociclib on autophagy and increased the intracellular cyclin D1 level.

Targeting the cell cycle is a rational approach for MCL therapy, as aberrant expression of cyclin D1 and dysregulation of CDK4 underlie cell cycle progression and MCL cell proliferation^32^. Cyclin D1, a major cell cycle regulator, is one of three unlinked D-type cyclin gene family members that also regulates transcription^33,34^. Cyclin D1 regulates the migration and invasion of MCL cells and can be viewed as a true operator in the pathogenesis of MCL^35^. By overexpressing cyclin D1, we found that overexpression of cyclin D1 also lowered the sensitivity of MCL cells to palbociclib; enhanced cell proliferation, migration, invasion, and autophagy; and inhibited cell death. In addition, Ambra1 expression was negatively correlated with cyclin D1 expression. This is also the first time that we report that Ambra1 and cyclin D1 play an important role in the mechanism of MCL sensitivity to palbociclib. Our observation also raises another possibility that the administration of CDK4/6 inhibitors (such as palbociclib), alone or in combination with cyclin D1 inhibitors, might be a possible therapeutic strategy for MCL. This possibility needs more exploration and confirmation in our future studies.

Importantly, Ambra1 expression is controlled by caspase-mediated cleavage and calpain-mediated degradation^36^. Recent research showed that RNF2 ubiquitylates and degrades Ambra1^37^. Moreover, noncoding RNAs (including circular RNAs and microRNAs) have been regarded as key regulators of Ambra1^38^. Through direct binding to the 3'-untranslated region of Ambra1 Mrna (Supplemet), circRNA-BCL2 may subsequently modify the production of miRNA-198 to control Ambra1 expression^38^. In addition, miR-200b inhibits autophagy and induces apoptosis by directly targeting the autophagy-associated gene Ambra1^39^. However, the exact genetic and epigenetic mechanisms that govern Ambra1 expression remain unclear, and future research is required to define how Ambra1 downregulation occurs in MCL. In addition, how Ambra1 affects cyclin D1 expression, migration, invasion, and apoptosis in tumor cells through autophagy and the molecular mechanism by which Ambra1 regulates cyclin D1 expression are not clear. Due to time and funding constraints, we are not currently able to solve these questions well. In the future, with sufficient time and funding, we will continue to explore how Ambra1 affects cyclin D1 expression, migration, invasion, and apoptosis in tumor cells through autophagy and the molecular mechanism by which Ambra1 regulates cyclin D1.

In conclusion, our study demonstrated that abnormal expression of the autophagy-related regulator Ambra1 affected the cyclin D1 level and interfered with the sensitivity of MCL cells to the CDK4/6 inhibitor palbociclib. Reversing the abnormal expression of Ambra1 could inhibit the occurrence and development of MCL. Our study provides a reference and basis for the clinical treatment of MCL and provides a new strategy for overcoming drug resistance in MCL.

## Supplementary Information

Supplementary Information.

## Data Availability

All data included in this study are available upon request by contact with the corresponding author.
