# Supplementary figures and images for: Ambra1 modulates the sensitivity of mantle cell lymphoma to palbociclib by regulating cyclin D1
Source: Sci Rep. 2023 May 24;13:8389. doi: 10.1038/s41598-023-35096-6 (PMC10209061; doi:10.1038/s41598-023-35096-6)

Figure1A

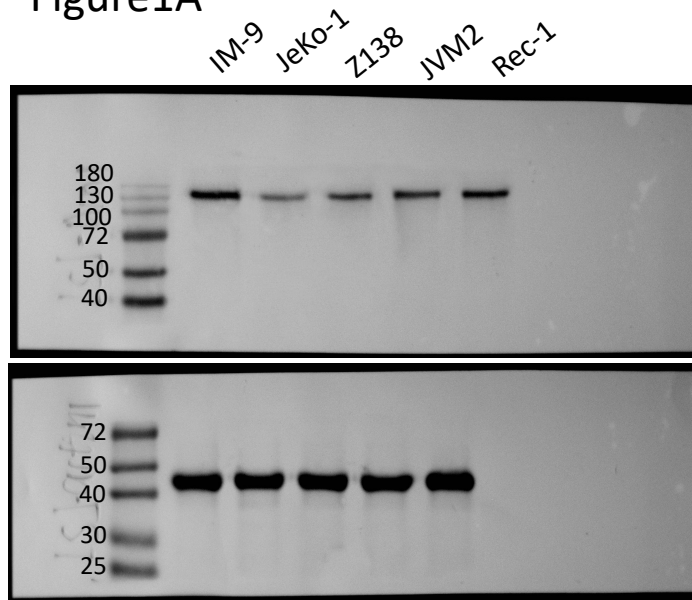

Figure1B

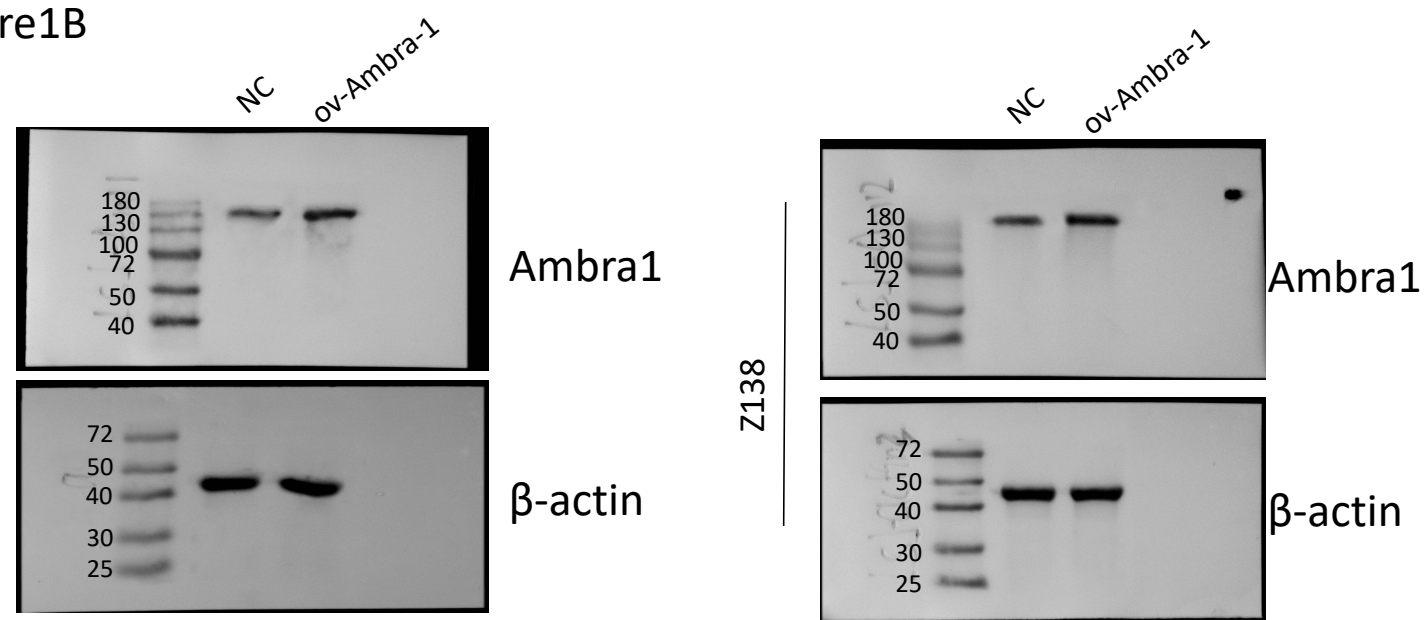

Figure1C

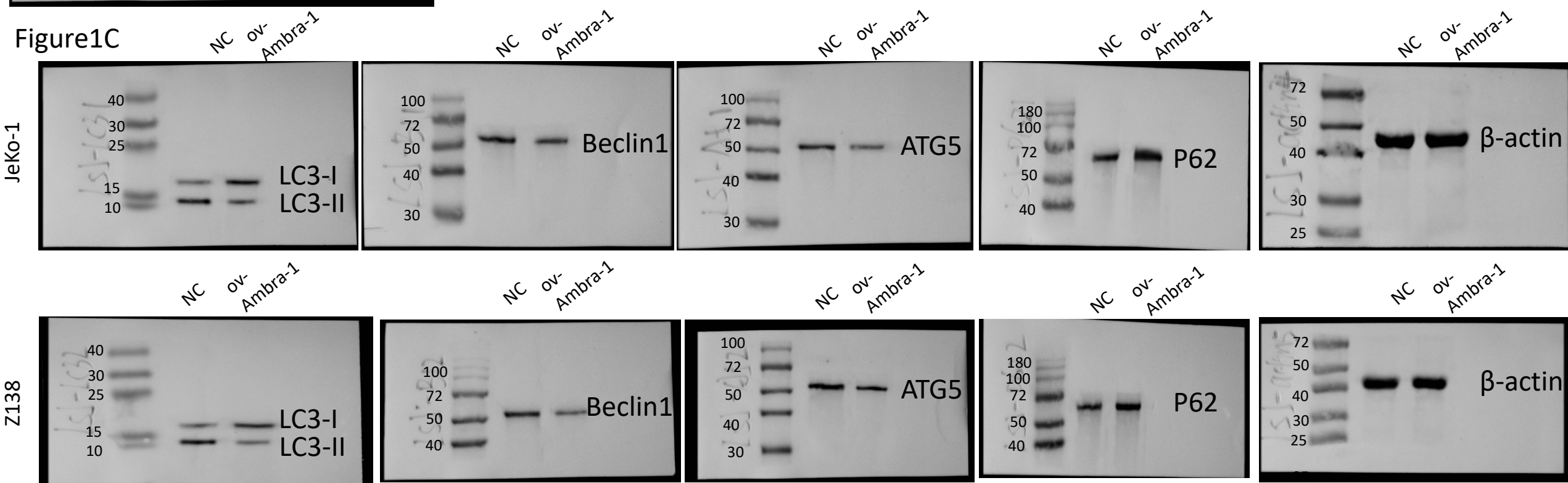

Figure2A

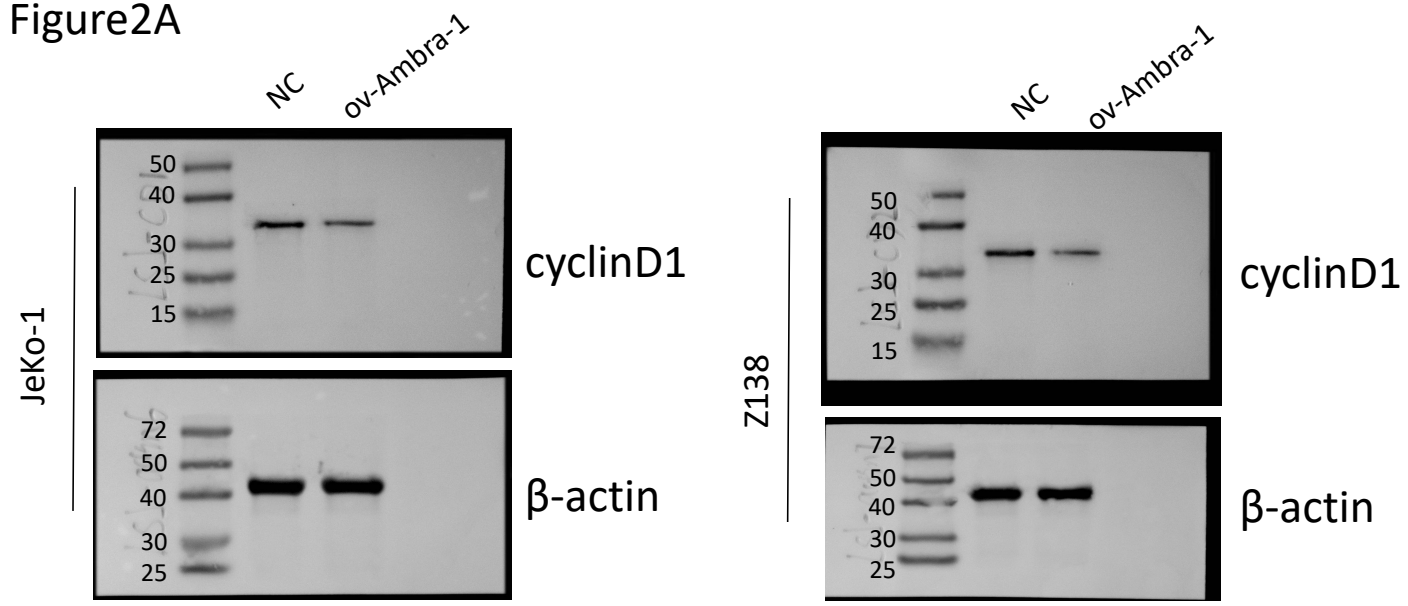

Figure3A

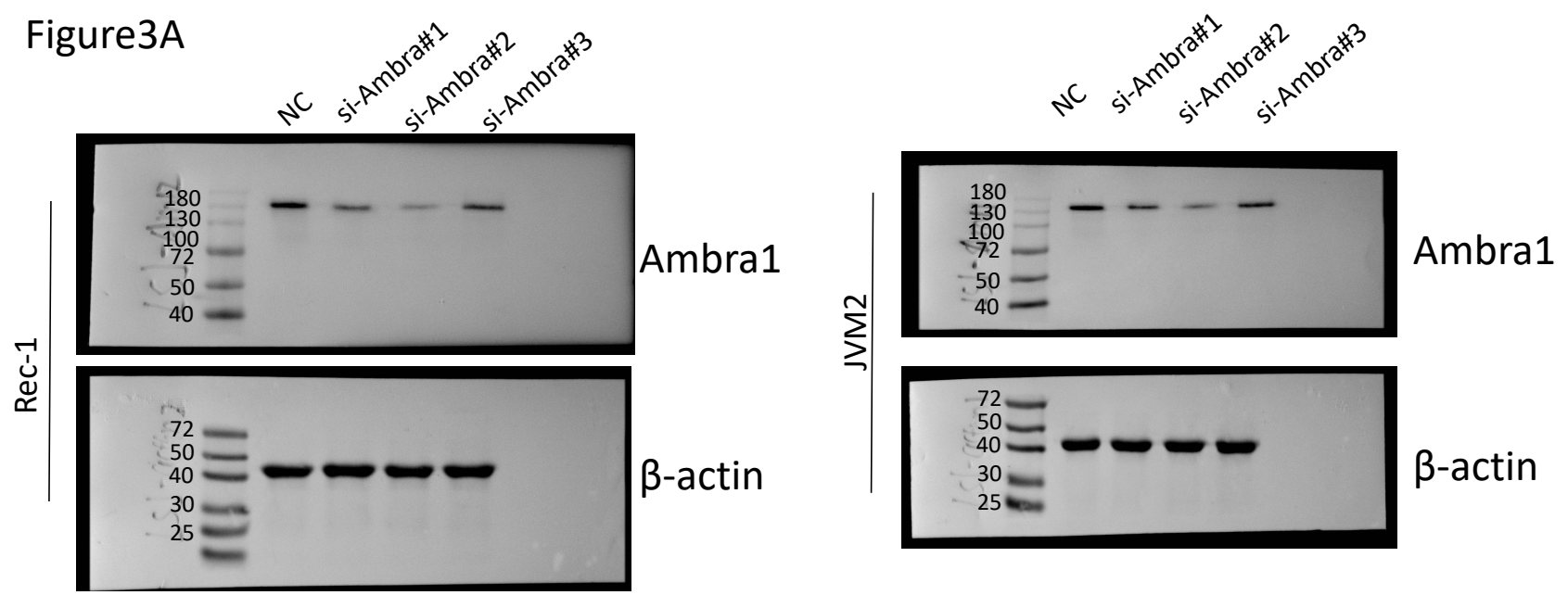

Figure4A

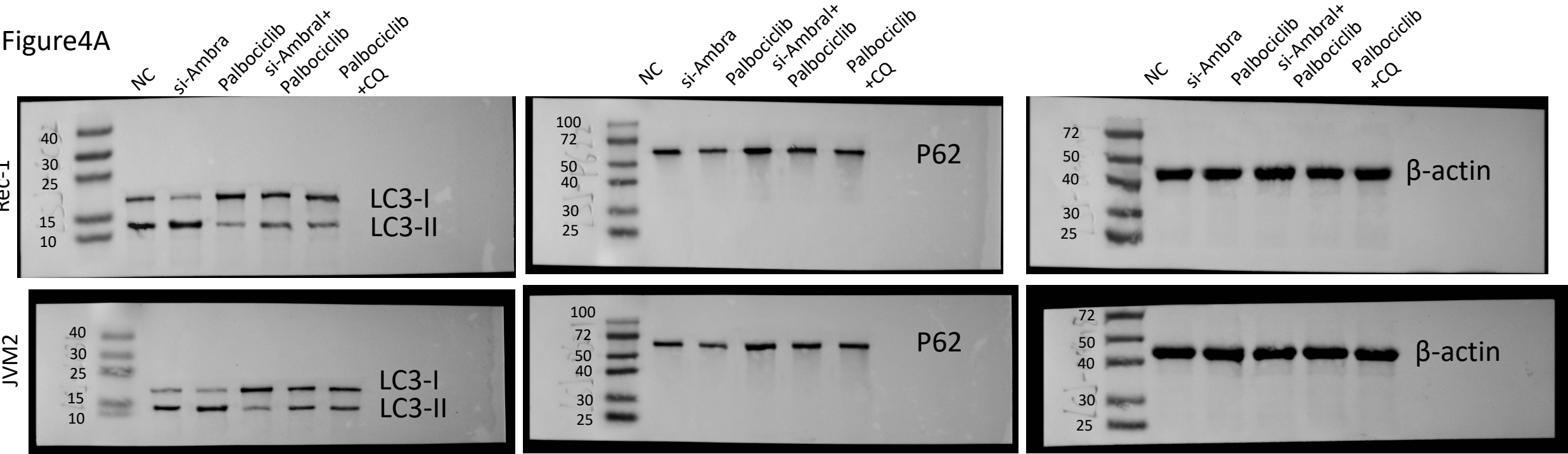

Figure4C

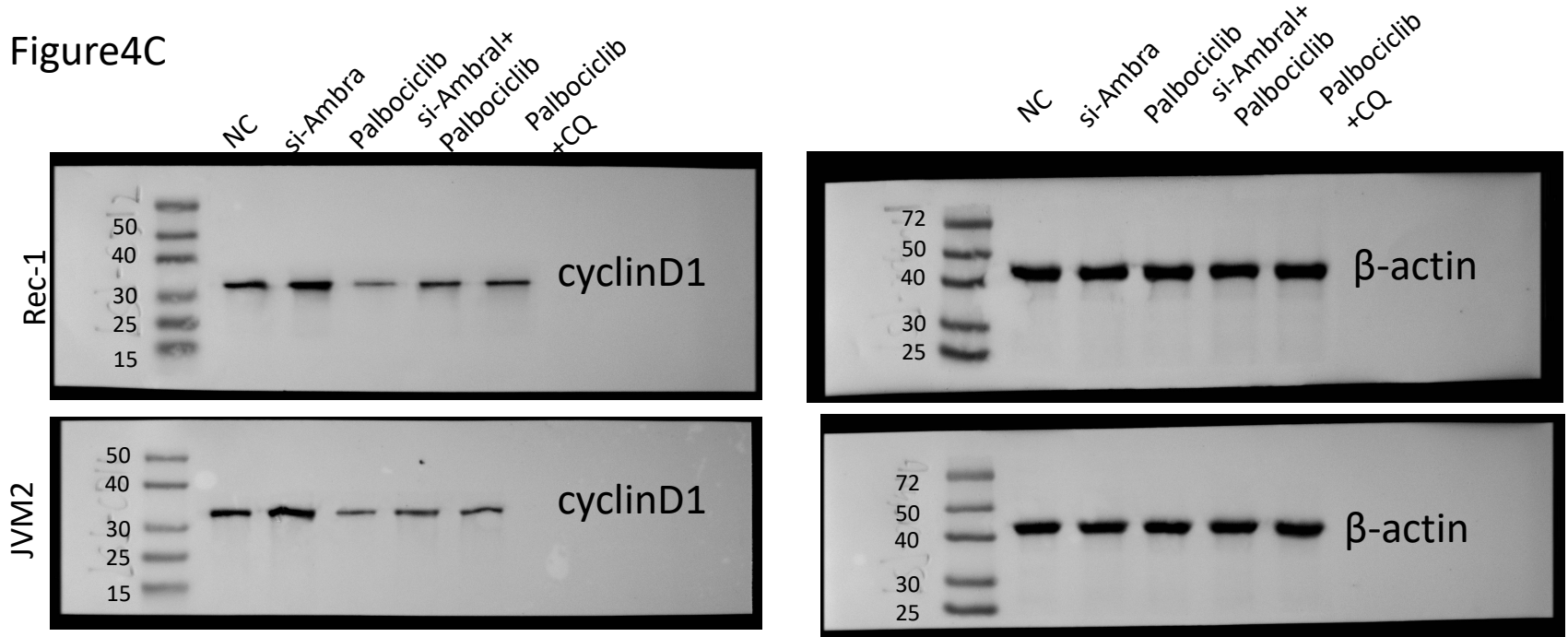

Figure5A

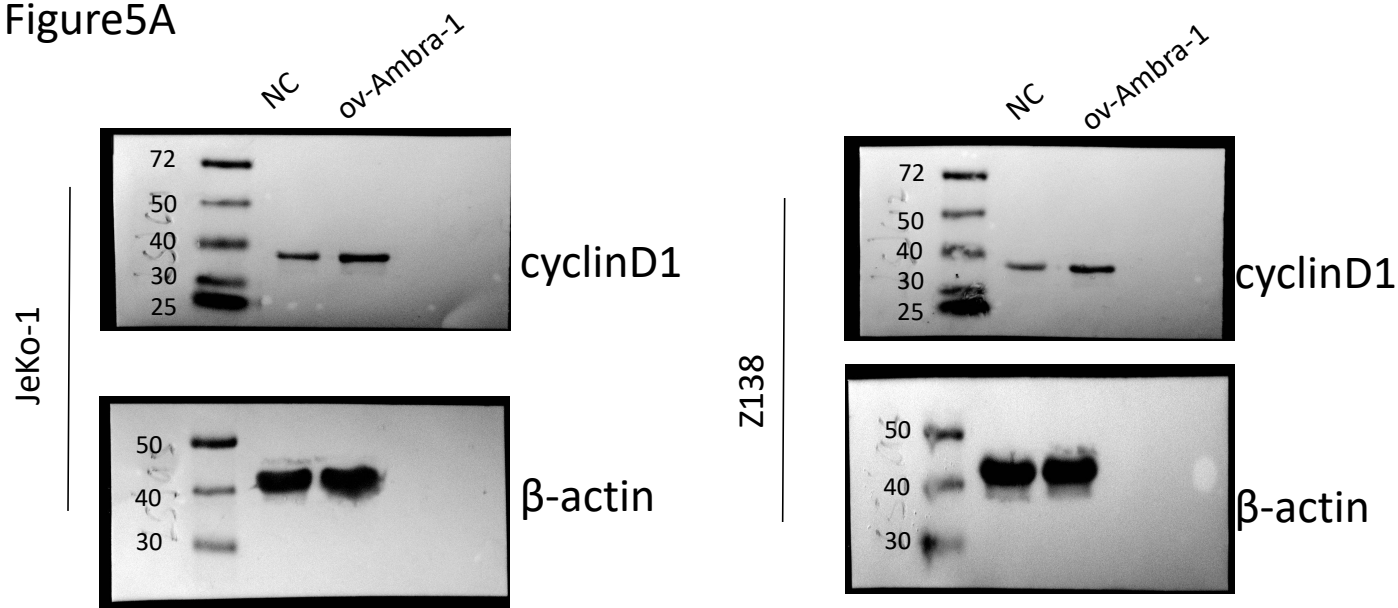

Figure5B

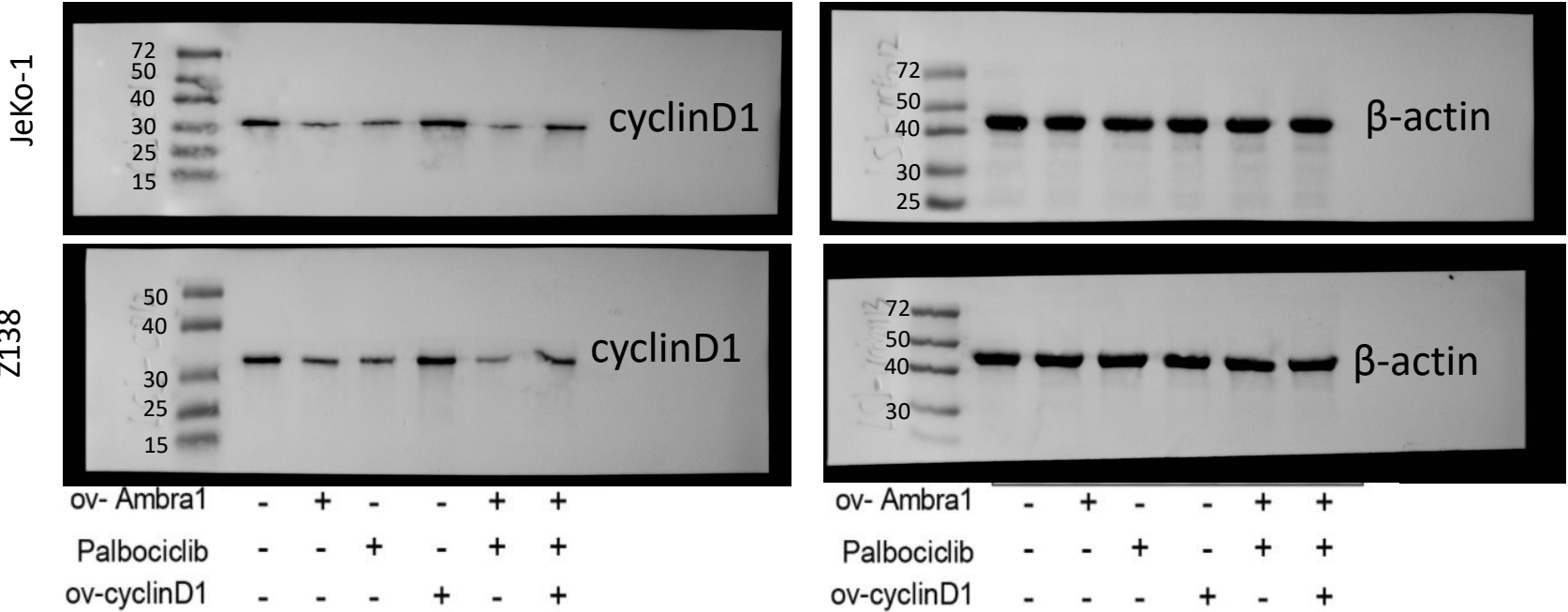

Figure5G

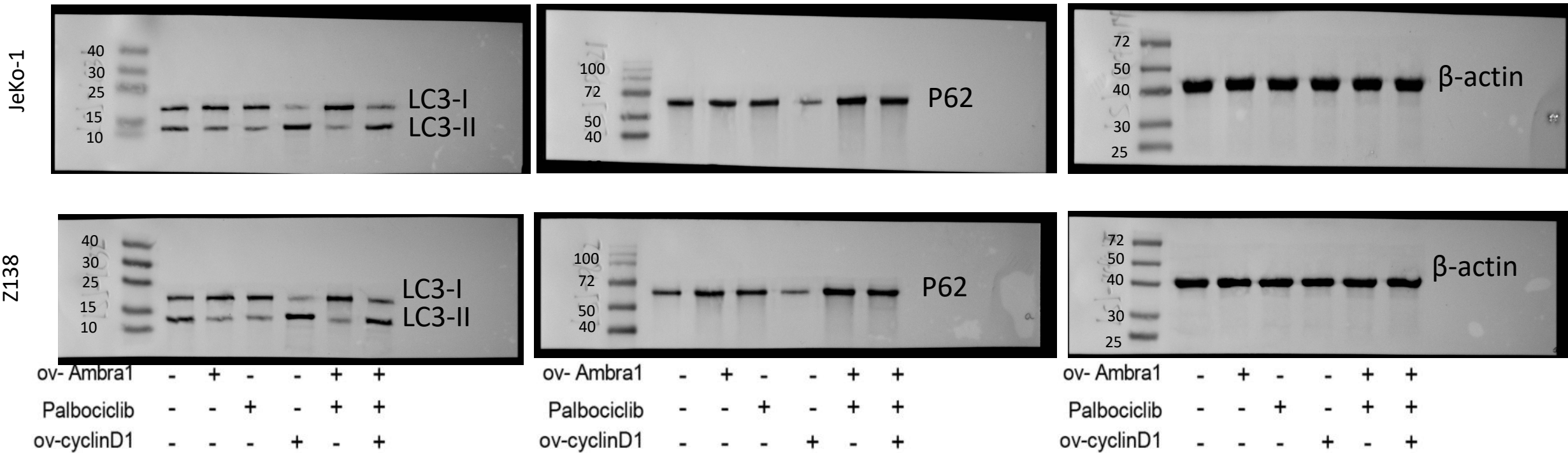

Figure6B

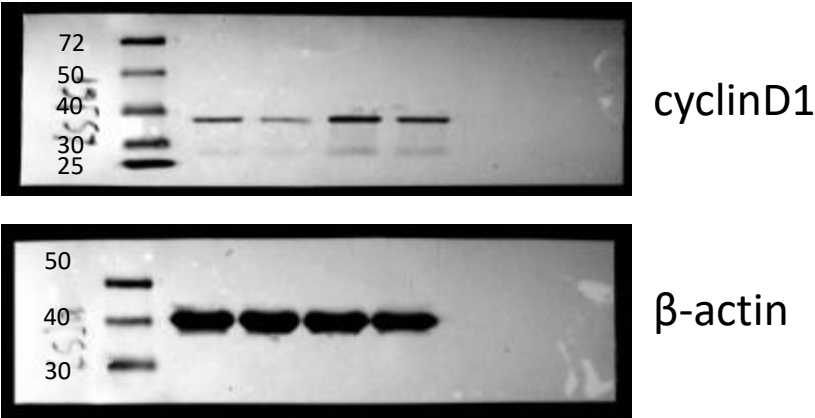

Supplement: Supplementary file 1 — Supplementary Information. [file 41598_2023_35096_MOESM1_ESM.pdf]
